# Comprehensive profiling of pathogenic germline large genomic rearrangements in a pan‐cancer analysis

**DOI:** 10.1002/1878-0261.13430

**Published:** 2023-04-12

**Authors:** Zhe Sun, Chujie Bai, Miaoyi Su, Haimeng Tang, Xiaoying Wu, Yue Wang, Hua Bao, Xunbiao Liu, Xue Wu, Yang Shao, Bei Xu

**Affiliations:** ^1^ The First Clinical Medical College Guangzhou University of Chinese Medicine Guangdong China; ^2^ Department of Bone and Soft Tissue Tumor, Key Laboratory of Carcinogenesis and Translational Research Peking University Cancer Hospital and Institute Beijing China; ^3^ Department of Radiation Oncology Guangqian Hospital Quanzhou China; ^4^ Geneseeq Research Institute Nanjing Geneseeq Technology Inc. China; ^5^ School of Public Health Nanjing Medical University China; ^6^ Department of Medical Oncology Zhongshan Hospital Shanghai China

**Keywords:** double‐hit hypothesis, large genomic rearrangement, next generation sequencing, pathogenic germline mutation

## Abstract

The presence of large genomic rearrangements (LGRs) has been heavily investigated in breast and ovarian cancer. However, correlations between LGRs and cancer types beyond these two have not been extensively profiled, likely due to the highly inefficient methods of detecting these types of alterations. This study utilized next‐generation sequencing (NGS) to analyze and classify the germline LGR profile in 17 025 cancer patients across 22 cancer types. We characterized newly identified LGRs based on predicted pathogenicity and took a closer look at genes that acquire both germline and somatic mutations within our samples. The detection method for LGRs was validated using droplet digital polymerase chain reaction (ddPCR) assay of commonly investigated LGR genes. In total, 15 659 samples from across 22 cancer types were retained for analysis after filtering. We observed that, in our cohort, the cancer types with the highest proportion of germline LGRs were ovarian cancer (4.7%), renal cell carcinoma (2.5%), breast cancer (2%), glioma (1.8%) and thyroid carcinoma (1.8%). Annotation of detected germline variants revealed several genes—*MSH2*, *FANCA* and *PMS2*—that contain novel LGRs. We observed co‐occurrences between germline LGRs in *MSH2* and somatic single nucleotide variants/insertion and deletions (SNVs/InDels) in *BRCA2*, *KTM2B*, *KDM5A*, *CHD8*, and *HNF1A*. Furthermore, our analysis showed that samples with pathogenic and likely pathogenic germline LGRs tended to also have higher mutational burden, chromosomal instability, and microsatellite instability ratio compared to samples with pathogenic germline SNVs/InDels. In this study, we demonstrated the prevalence of pathogenic germline LGRs beyond breast and ovarian cancer. The profiles of these pathogenic or likely pathogenic alterations will fuel further investigations and highlight new understanding of LGRs across multiple cancer types.

## Graphical abstract

Germline large genomic rearrangements (LGRs) are involved in many cancer types. *RB1*, *MSH2*, *FANCA* and *PMS2* have a significantly higher ratio of germline LGRs compared to germline single nucleotide variants/insertion and deletions (SNVs/InDels). Germline LGRs in *MSH2* co‐occur with many somatic mutations. Patients with germline LGRs have higher chromosomal instability and microsatellite instability than patients with germline SNVs/InDels or without germline mutations.

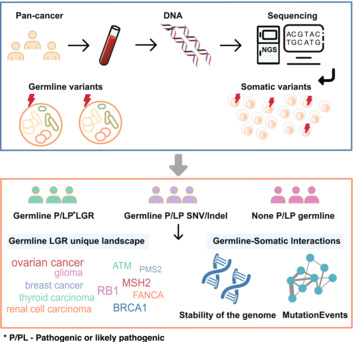

**Abbreviations**
CIN: chromosomal instability number
CNV: copy number variation
ddPCR: Droplet Digital Polymerase Chain Reaction
DDR: DNA damage repair
FA: Fanconi anemia
FFPE: formalin‐fixed paraffin‐embedded
HR: homologous recombination
IGV: integrative genomics viewer
LGR: large genomic rearrangement
MLPA: multiplex ligation‐based probe amplification
MMR: mismatch repair
MSI: microsatellite instability
NGS: next generation sequencing
SNV/InDel: single nucleotide variant/insertion and deletion
TMB: tumor mutation burden
VAF: variant allele frequency
WGD: whole genome duplication

## 1 Introduction

In general, large genomic rearrangements (LGRs) are defined as deletions or duplication events in the scale of one or more exons of a gene and can usually span between hundreds to millions of base pairs. The presence of such rearrangements can potentially lead to the development of neoplasms through inactivation of tumor suppressor genes. The most well‐characterized examples of LGRs are on *BRCA1/2*, both of which have been profiled in multiple solid tumors [1, 2, 3, 4]. The KOHBRA study, published in 2011, estimated *BRCA1/2* LGRs to be high (~20–23%) in the Korean hereditary breast or ovarian cancer populations [5]. In addition to *BRCA1*/2, *RB1* LGRs have also been identified in certain populations with retinoblastoma [6]. These mutations have been reported to vary in proportion based on population [1, 3, 4, 6].

Conventional methods of detecting mutations are unable to accurately identify LGRs due to the size of the alterations, however, copy number sensitive methods such as next generation sequencing (NGS) or multiplex ligation probe amplification (MLPA), the latter of which is considered by many to be the gold standard of LGR detection [2, 4, 7, 8]. In this study, we use targeted NGS to detect LGRs in multiple cancer types in an Asian population. Called variants were validated using droplet digital PCR (ddPCR) [9, 10, 11] to probe three previously defined LGR genes and compare with our detected variants. The ddPCR method is a relative new comparing to MLPA but also has been proven to be sensitive and accurate in LGR detection in previous studies [9, 10, 12, 13].

We characterized pathogenic germline LGRs present in multiple cancer types. To the best of our knowledge, this detailed study is the first investigation into LGRs in a pan‐cancer setting. We also effectively profiled and classified novel detected pathogenic or likely pathogenic germline LGRs. Through our descriptive study, we present an overview of the proportions of germline LGRs in a Chinese population.

## 2 Materials and methods

### 2.1 Sample selection

**Fig. 1:**
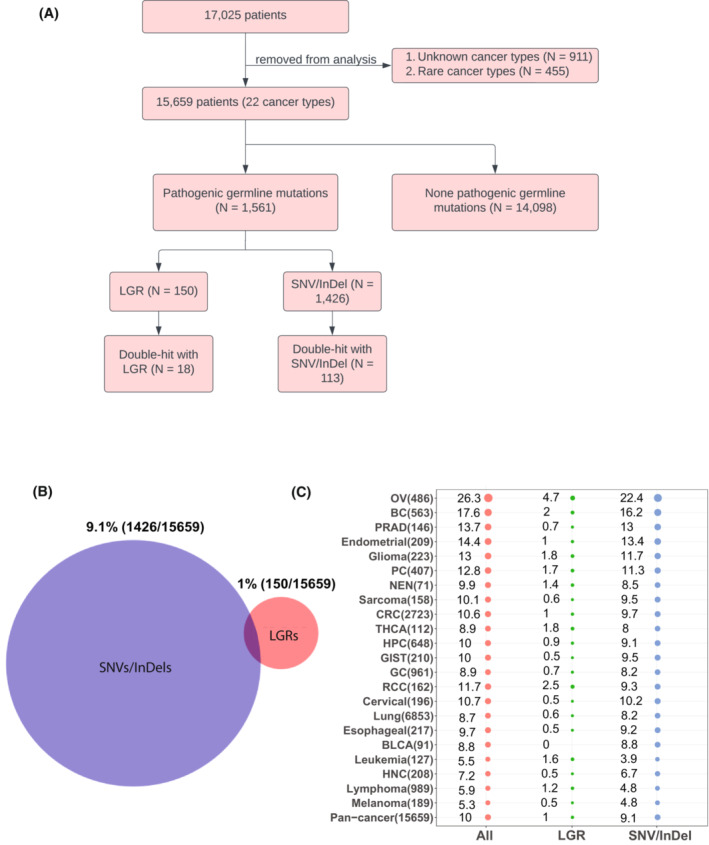
Cohort characteristics. (A) Flow chart of patient selection process for inclusion into study. (B) Venn diagram showing the total sample numbers and overlap in samples from each group. Red circle indicates the proportion of patients with pathogenic germline LGRs and blue circle indicates the proportion of patients with pathogenic germline SNVs/InDels, and (C) proportion of pathogenic germline LGRs and pathogenic germline SNVs/InDels across 22 cancer types. The circle size denotes proportion size, with a larger circle meaning higher proportion. Number in parentheses following the cancer type indicates the number of patients from each category. BC, breast cancer; BLCA, bladder cancer; CRC, colorectal cancer; GC, gastric cancer; GIST, gastrointestinal stromal tumor; HNC, head and neck cancer; HPC, hepatocellular cancer; NEN, neuroendocrine tumors; OV, ovarian cancer; PC, pancreatic cancer; PRAD, prostate adenocarcinoma; RCC, renal cell carcinoma; THCA, thyroid cancer.

Initially, 17 025 patients across 22 cancer types were consecutively collected from multiple institutions and not pre‐selected with National Comprehensive Cancer Network eligibility for germline genetic testing. Patient eligibility was then determined by two steps: (a) the cancer type of the patient was not unspecified or a rare cancer; (b) a tumor sample and a normal sample (whole blood) were obtained from each patient. Figure 1A,B shows the flow chart of patient selection and the relative proportions of patients with pathogenic germline LGRs, SNVs/InDels or both. Figure 1C shows the proportion of pathogenic germline LGRs, SNVs/InDels and all pathogenic germline mutations in the cohort across 22 cancer types, with the last row summarizing all mutations into a pan‐cancer dataset with a total proportion of 10% pathogenic germline mutations (1% LGR, 9.1% SNV/InDel). Patients were informed of research intent and permission for data collection was obtained from all subjects through written consent. This study was approved by Medical Ethics Committee of Nanjing Geneseeq Medical Laboratory (Ethics Committee Register Number: NSJB‐MEC‐2022‐04) and conforms to the Declaration of Helsinki.

### 2.2 LGR detection

Extraction of DNA and sequencing libraries was conducted according to previous literature. Briefly, genomic DNA was extracted from fresh tumor or formalin‐fixed paraffin‐embedded (FFPE) tissue samples and normal control samples were obtained from peripheral white blood cells. Customized xGen lockdown probes (Integrated DNA Technologies, Diego, CA, USA) were designed according to instructions provided by provider. DNA libraries were quantified using qPCR (KAPA Library Quantification kit, KAPA Biosystems, Wilmington, MA, USA) and fragment sizes were calculated using the Bioanalyzer 2100 instrument (Agilent Technologies, Santa Clara, CA, USA).

A total of 437 cancer related genes were targeted for sequencing by the Geneseeq Prime™ panel (Nanjing Geneseeq Technologies Inc., Nanjing, Jiangsu, China). All samples were sequenced on the Hiseq 4000 instrument (Illumina, San Diego, CA, USA). We removed low quality regions on sequenced reads with trimmomatic (v0.39, https://www.anaconda.org/bioconda/trimmomatic), which were then aligned to the hg19 human reference genome using bwa (Burrows‐Wheeler Aligner v0.7.12, https://www.github.com/lh3/bwa). Processing of the trimmed and aligned reads, including deduplication, local realignment around indels, and base quality score recalibration, was performed using recommended tools in genome analysis toolkit v3.4.0 (https://www.github.com/broadinstitute/gatk/releases). Germline and somatic mutations were called using GATK haplotypecaller and Mutect2 respectively.

A list of 135 cancer predisposition genes (described in Section 2.4) were analyzed to determine pathogenic germline LGRs. We calculated the exon amplifications and deletions based on the CNVs in combination with SNP loci status, that is, either homozygous or heterozygous. The quality control for each CNV was calculated using the variance to previous (VP) for the log2ratio of each target region using an in‐house algorithm. This LGR detection method was validated by ddPCR procedures described in Section 2.3. A double‐hit event is defined as simultaneous occurrences of germline mutations (1st hit) and somatic mutations (2nd hit) on the same gene in a sample.

### 2.3 Validation of LGR detection method

To validate the LGR mutation calls, we carried out ddPCR for BRCA1/2 and RB1 on 15 samples. The methods and procedures for conducting ddPCR have been described in an earlier publication and were adapted here [14]. Detection of variants in BRCA1/2 and RB1 was performed on the QX200 ddPCR system (Bio‐Rad, Hercules, CA, USA). Primers and probes were customized and synthesized by Integrated DNA Technologies (IDT). Each reaction was set up containing 50 ng genomic DNA, 9 pmol of each primer, 5 pmol of each probe, and 10 μL of 2× ddPCR Supermix for probes (No dUTP) (Bio‐Rad) in a 20 μL reaction volume. The following PCR conditions were used: (a) an initial activation step at 95 °C for 10 min; (b) followed by 45 cycles of denaturation at 94 °C for 30 s and annealing/elongation at 60 °C for 1 min; (c) followed by a final elongation at 60 °C for 5 min. PCR temperature ramp rate was set at 2 °C·s^−1^ for every step. Each reaction was set up following the manufacturer's instructions and containing 50 ng genomic DNA. PCR was carried out following the manufacturer's instructions for each commercial assay. PCR products were then subjected to analysis by the QX‐200 droplet reader and QuantaSoft™ Analysis Software (Bio‐Rad). NA18535 was used as a baseline for mutation detection. If at least three positive droplets were detected in a sample and the total number of positive droplets exceeds 3 times the average number of positive droplets in the five replicates of NA18535, we deem that sample as being positive. We used CNV detection to deduce genomic rearrangement of exons in *BRCA1/2* and *RB1*. To control for potential errors introduced during the detection process, we included a reference gene (*RPP30*) with high copy number stability to act as a negative control. The resulting exon‐level CNV calls were then compared with NGS detected CNVs from the same sample to determine sensitivity, specificity, and accuracy as shown in the results.

### 2.4 Annotation of pathogenic germline mutations

A list of 135 cancer predisposition genes were summarized according to literature and consensus (National Comprehensive Cancer Network Guidelines, OncoKB, ClinVar, [15, 16, 17]) to define pathogenic germline LGRs and SNVs/InDels. Pathogenic germline SNVs/InDels was annotated using 4 commonly referred human population databases (ESP6500, ExAC, gnomAD, 1000 Genome Project, https://www.internationalgenome.org/). We then performed automated scoring and predictive interpretation using several softwares (polyphen [18], sift [19], cadd [20], gerp++ [21], Likelihood Ratio Test, and MutationTaster [22]) to estimate the risk of each SNV/InDel for causing cancer to develop. For the sake of concision, pathogenic germline mutations also include likely pathogenic mutations (LGRs or SNVs/InDels), and LGRs are all pathogenic or likely pathogenic germline LGRs in this paper.

### 2.5 Statistical analysis

Statistical tests were performed in r v4.1.1 (https://www.cran.r-project.org/). Significant differences between boxplots were calculated using the Mann–Whitney *U* test with a significance cut‐off at *P* ≤ 0.05. Proportion of groups in categorical variables were compared using Fisher's test. Differences were determined to be significant if the *P* ≤ 0.05. In this study, tumor mutation burden (TMB) was defined as the number of non‐synonymous mutations per mega base of sequence in tumor sample. Chromosomal instability (CIN) was calculated in tumor samples based on the percentage of total segments with copy number variation. Whole genome duplication (WGD) in tumor samples was calculated using segment copy numbers. If the copy number of a segment is greater than two, we denote that segment as having a duplication event. If the total length sum of segments with duplication events is greater than 50% of the total chromosomal length of a sample, we define that sample as being WGD. Whole genome duplication ratio (WGD ratio) was subsequently defined as the proportion of samples in a group that is WGD.

## 3 Results

### 3.1 Patient characteristics

In this study, we obtained samples from 17 025 cancer patients across more than 22 cancer types. Of those, 15 659 patients were selected for further analysis. Upon investigating the presence of potentially cancer related variants in our cohort, we found 1561 patients with pathogenic germline mutations (Fig. 1A), among which 150 patients had LGRs (15 of these patients also had SNVs/InDels; Fig. 1A,B). We then observed 113 and 18 cases, respectively, of double‐hit gene events in each group of patients. Arranging the patients based on cancer type reveals that the highest proportion of germline LGRs can be found in ovarian cancer (4.7%), renal cell carcinoma (2.5%), breast cancer (2%), thyroid cancer (1.8%) and glioma (1.8%) (Fig. 1C).

Patient characteristics are summarized in Table 1. Patients were separated into three groups base on mutational status: without pathogenic germline mutations [“Without”], *N* = 14 098; with only germline LGRs [“LGR”], *N* = 135; with only germline SNVs/InDels [“SNV/InDel”], *N* = 1411. Patients with both germline LGRs and germline SNVs/InDels (*N* = 15) have been excluded for statistical analysis due to the uncertainty of the classification in regards to comparing the difference of features between germline LGRs and germline SNVs/InDels.

**Table 1:** Patient characteristics.

	Without pathogenic germline mutations (*N* = 14 098)	Pathogenic germline SNVs/InDels (*N* = 1411)	Pathogenic germline LGRs (*N* = 135)	*P* value
Age at diagnosis				
≤ 60	6126 (43.5%)	674 (47.8%)	56 (41.5%)	< 0.001
> 60	5871 (41.6%)	518 (36.7%)	40 (29.6%)	
Unknown	2101 (14.9%)	219 (15.5%)	39 (28.9%)	
Median(range)	60 (2–94)	58 (3–90)	57 (13–85)	
Sex				
Female	6444 (45.7%)	722 (51.2%)	70 (51.9%)	< 0.001
Male	7654 (54.3%)	689 (48.8%)	65 (48.1%)	
Stage				
I	107 (0.8%)	13 (0.9%)	0 (0%)	< 0.001
II	90 (0.6%)	24 (1.7%)	5 (3.7%)	
III	232 (1.6%)	31 (2.2%)	5 (3.7%)	
IV	1789 (12.7%)	178 (12.6%)	17 (12.6%)	
Unknown	11 880 (84.3%)	1165 (82.6%)	108 (80.0%)	
Number of cancers				
Single cancer	13 864 (98.3%)	1369 (97.0%)	132 (97.8%)	0.004
Multiple cancer	234 (1.7%)	42 (3.0%)	3 (2.2%)	
Family history				
With	3363 (23.9%)	386 (27.4%)	30 (22.2%)	0.465
Without	1005 (7.1%)	99 (7.0%)	9 (6.7%)	
Unknown	9730 (69.0%)	926 (65.6%)	96 (71.1%)	

When comparing proportions of age, sex, cancer stage, and number of patients with multiple cancer types, Fisher's exact test showed significant differences between the three groups (age *P* < 0.001, sex *P* < 0.001, stage *P* < 0.001, Number of cancers *P* = 0.004). Comparison between every two groups showed that “SNV/InDel” group had a significant higher proportion of females (*P* < 0.001), patients ≤ 60 years old (*P* < 0.001) and patients with multiple cancers (*P* < 0.001) comparing to “Without” group; both “SNV/InDel” group and “LGR” groups had significantly more patients in early stage comparing to patients without germline mutations; no significant difference were found between “SNV/InDel” group and “LGR” group in terms of these clinical features (Table S1).

### 3.2 LGRs calling validation

Prior to categorizing the presence of LGRs across cancer types, to validate the LGR calling method, we used a previously reported copy number sensitive method with ddPCR [9, 10, 11] to detect *BRCA1/2* LGRs in 7 samples and *RB1* LGRs in eight samples (Fig. S1A). The results were then compared to the alterations identified through our targeted NGS approach to determine sensitivity, specificity, and accuracy. In *BRCA1* (*N* = 161 exons) and *BRCA2* (*N* = 196 exons), 100% sensitivity was achieved respectively at 98.25% specificity (accuracy 98.76%) and 86.60% specificity (accuracy 86.73%), with a total of 159 concordant exon calls for *BRCA1* and 170 concordant exon calls for *BRCA2* (Fig. S1B). On the other hand, LGRs detected on *RB1* using ddPCR and NGS methods had an exon call accuracy of 97.22% (*N* = 216 exons, Sens: 95.00%, Spec: 100.00%, 210 concordant exon calls; Fig. S1B). Figure S1C–F shows the locations of large genomic rearrangements through the Integrative Genomics Viewer (igv) v2.14.0 (Coralville, IA, USA).

### 3.3 Characterization of LGRs

**Fig. 2:**
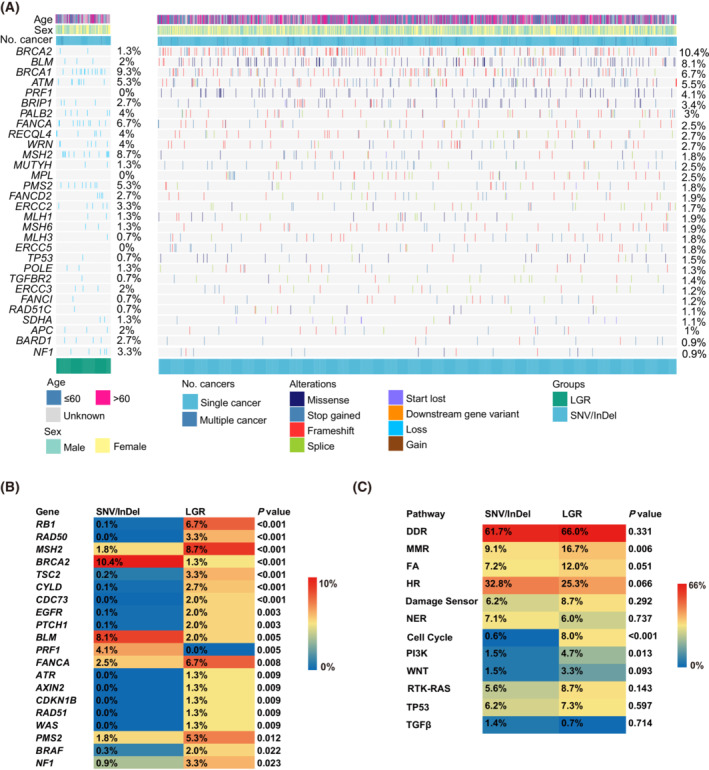
Mutational landscape of pathogenic germline LGRs and SNVs/InDels. (A) Oncoprint describing the mutational landscape of top 30 most mutated genes. Genes were sorted by mutational prevalence in pathogenic germline SNVs/InDels. Patient information is displayed on top. (B) Comparison of the proportion of gene frequency between LGRs and SNVs/InDels. *P* values were calculated using Fisher's test and significance was set at < 0.05. Genes were ordered based on *P* values. (C) Comparison of the proportion of pathway frequency between LGRs and SNVs/InDels. *P* values were calculated using Fisher's test and significance was set at < 0.05. DDR, DNA damage repair; FA, Fanconi Anemia; HR, homologous repair; MMR, mismatch repair; NER, nucleotide excision repair.

To illustrate the mutational landscape in the cohort, we summarized the prevalence of germline LGRs in the oncoprint (Fig. 2A). Of the top genes with germline LGRs, we observed the highest proportion of germline SNVs/InDels in *BRCA2* (10.4%), *BLM* (8.1%), *BRCA1* (6.7%), *ATM* (5.5%), and *PRF1* (4.1%). The proportion of samples with *BRCA1* LGR and *BRCA2* LGR are in line with previous publications [8, 23].

Comparisons of germline LGRs and germline SNVs/InDels in each of the top genes with LGRs show that the LGR rate is significantly higher than the SNV/InDel rate in *RB1* (6.7% vs 0.1%, *P* < 0.001), *MSH2* (8.7% vs 1.8%, *P* < 0.001), *FANCA* (6.7% vs 2.5%, *P* = 0.008), *PMS2* (5.3% vs 1.8%, *P* = 0.012; Fig. 2B). Pathogenic germline mutations, both SNVs/InDels and LGRs, were observed to occur frequently on the DNA damage repair (DDR) and homologous recombination (HR) pathways. Germline LGR distributions also tend to appear in cell cycle, mismatch repair (MMR), Fanconi anemia (FA), and PI3K pathways, while the germline SNV/InDel distributions have no significant gathering in any other pathways except DDR and HR, which indicates that in comparison to germline SNVs/InDels, germline LGRs occur more randomly in different pathways, resulting in a diverse and unique mutational landscape (Fig. 2C).

**Fig. 3:**
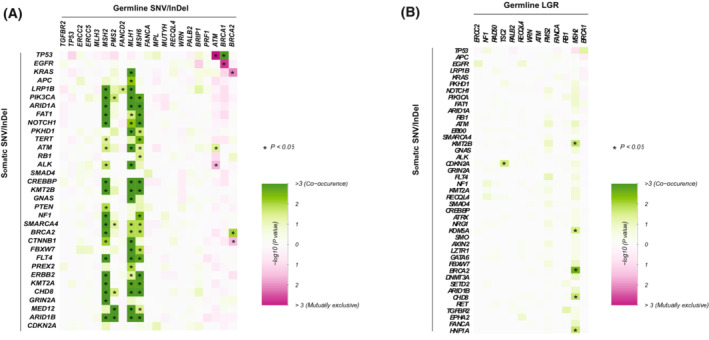
Co‐occurrences of pathogenic germline mutations (LGR and SNVs/InDels separately) with somatic SNVs/InDels. Presence of co‐occurrences between (A) germline SNVs/InDels vs somatic SNVs/InDels and (B) Germline LGRs vs somatic SNVs/InDels. Asterisks (*) indicate statistical significance as determined by a cut‐off of *P* < 0.05 using pairwise Fisher's exact test.

Germline SNVs/InDels in *MSH2/6*, *MLH1* and *PMS2* have significantly higher frequency of co‐occurrence with somatic mutations in a range of oncogenes and tumor suppressor genes (*P* < 0.05; Fig. 3A). *MSH2/6, MLH1* and *PMS2* are MMR genes and their germline variants have also been found to be cancer predisposing in previous studies [24]. Germline SNV/InDels in *ATM* is mutually exclusive with *TP53* somatic mutations (*P* < 0.05; Fig. 3A), which is in accordance with previous study and might be due to the epistatic relationship between *ATM* and *TP53* genetic alterations [25]. In germline LGRs, we found that *MSH2* gene has co‐occurrences with *BRCA2，KMT2B, KDM5A, CHD8, HNF1* somatic SNVs/InDels, and germline LGRs in *TSC2* and somatic SNVs/InDels in *CDKN2* display significant trend of co‐occurrences (*P* < 0.05 for all genes; Fig. 3B).

### 3.4 LGRs do not aggregate on specific exons

To determine whether LGRs preferentially aggregates on certain exons, we examined the exon‐level mutation counts for 4 genes with high LGR prevalence, that is, *BRCA1*, *MSH2*, *FANCA* and *ATM*. First, we observed a prominent accumulation of germline SNVs/InDels on Exon 11 of *BRCA1* (Fig. S2A) and Exon 62 of *ATM* (Fig. S2D). Next, although there are noticeable differences between mutant counts on exons in germline LGRs and SNVs/InDels genes, we did not find any mutational hotspots on the exons of genes with LGRs (Fig. S2A–D). Therefore, we deduce that LGRs do not specifically develop on certain exons and thus will require highly stringent parameters in terms of panel design.

### 3.5 Sample level metrics of pathogenic germline mutations

**Fig. 4:**
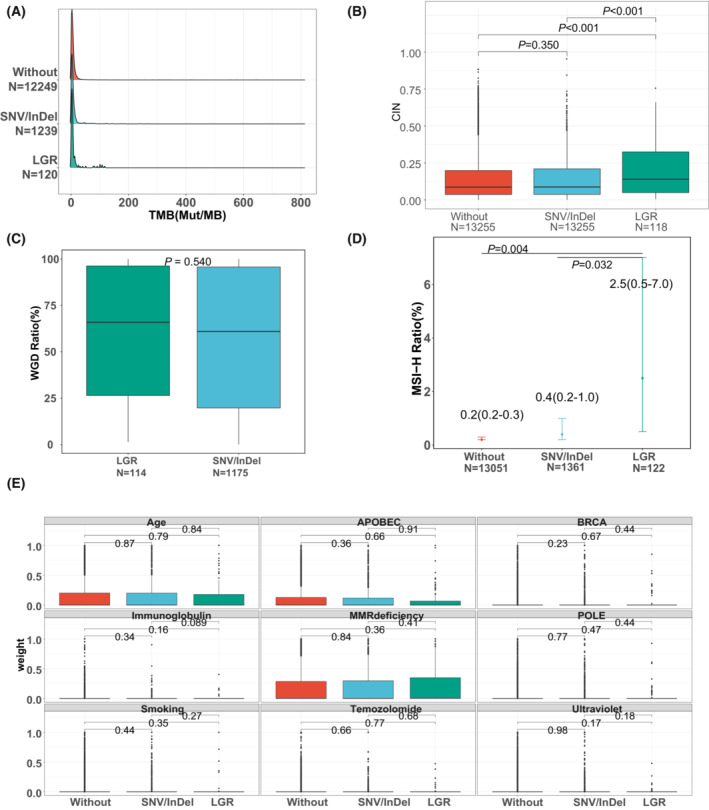
Comparisons of TMB, CIN, WGD, MSI and mutational signatures between patients grouped by pathogenic germline mutations. (A) Density distribution of tumor mutation burden (TMB) in each grouping scheme (Without: patients without pathogenic germline mutations; SNV/InDel: patients with only germline SNVs/InDels; LGR: patients with only germline LGRs). TMB is defined as the number of non‐synonymous mutations (Mut) per million bases of sequence (MB). Other considered metrics include (B) chromosomal instability number (CIN), (C) whole genome duplication (WGD), (D) microsatellite instability—high (MSI‐H), and (E) mutational signatures. Comparisons of CIN and WGD between groups were made with the Wilcoxon ranked‐sum test and significance was determined at *P* < 0.05. Comparison of MSI‐H between groups was made with Fisher's test with significance set at *P* < 0.05. Error bars indicate min/max in (B), (C) and (E); the error bar in (D) indicates 95% confidence interval.

To examine the effect of LGRs on the stability of the genome, we performed statistical analysis on 4 sample‐level metrics obtained from all groups. Figure 4A shows a density distribution of TMBs within each group. From this, we observed that the LGR group has a wider range of TMB values than other groups. Those with LGRs, in comparison to SNVs/InDels, have higher CIN (*P* < 0.001; Fig. 4B). Interestingly, the CIN of samples without pathogenic germline mutations and those with SNVs/InDels were statistically indistinguishable. In terms of WGD events, we did not observe significant differences between the groups with LGRs and the group with SNVs/InDels (Fig. 4C). Also, all patients with LGRs have a higher ratio of samples with high microsatellite instability status (MSI‐H) than those without these mutations (LGR vs Other: *P* = 0.032; LGR vs Without: *P* = 0.004; Fig. 4D). Considering the trends of higher ratios of MSI‐H and CIN in LGR patients might be caused by more frequent MMR‐gene mutations in LGR patients, we further compared these features in patients with only MMR‐gene mutations and found that the trends of higher ratios of MSI‐H and CIN in LGR patients did not appear in patients with only MMR‐gene mutations (Fig. S3) but persist after removing patients with MMR genes (Fig. S4). Finally, Fig. 4E displays the observed mutational signature landscape of our samples.

### 3.6 Presence of double‐hit events on genes

To determine whether presence of double‐hit events is correlated to any specific LGRs, we recorded the difference in proportion of double‐hit events occurring in patients with LGRs and patients with SNVs/InDels.

**Fig. 5:**
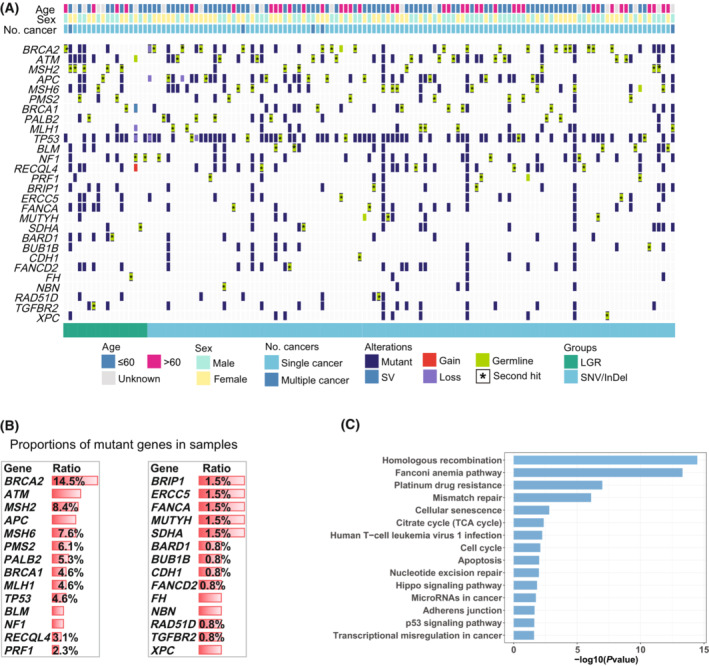
Genomic landscape of patients with double‐hit events on genes with pathogenic germline LGRs or SNVs/InDels. (A) Oncoprint showing the mutational landscape of the 28 genes with detected double‐hit events. Patient information is displayed on top. Gene names are ordered by the prevalence of double‐hit events. (B) Proportion of genes in double‐hit events. (C) Significantly mutated pathways with double‐hit events based on KEGG pathway enrichment.

One hundred and thirty‐one patients were discovered to have double‐hit events (8.4%; 18 patients with germline LGRs for the 1st hit, 113 patients with germline SNVs/InDels for the 1st hit, Fig. 5A). In our cohort, the genes with the highest proportion of double‐hit events are *BRCA2* (14.5%), *ATM* (9.2%), *MSH2* (8.4%), *APC* (7.6%), and *MSH6* (7.6%) (Fig. 5B). Furthermore, pathway‐level analysis of genes experiencing double‐hit events revealed that a significant proportion of these genes revolve around the homologous recombination pathway and Fanconi anemia pathway (Fig. 5C). No distinct differences could be found between the patients with double‐hit events on genes with LGRs and those with double‐hit events on genes with SNVs/InDels in terms of mutant count, TMB, CIN, WGD, or mutational signatures (Fig. S5A–E). However, mutant count, TMB, and CIN were observed to be generally higher in patients with LGRs than patients with SNVs/InDels, albeit not to a statistically significant degree.

## 4 Discussion

In this retrospective observational study, we sought to classify pathogenic germline LGRs detected using an NGS approach by using a multitude of genomic metrics. We verified our LGR detection method by comparing with ddPCR calls from three genes (*BRCA1*, *BRCA2*, and *RB1*) with well‐defined LGR regions. We found a high degree of concordance between the two methods, which proves the validity of this NGS method in LGR detection。

We believe that our cohort of 22 cancer types can sufficiently represent the array of pathogenic germline LGRs as detected in a real‐life population. The top five cancer types of germline LGRs in the cohort of 15 659 patients are ovarian cancer (4.7%), renal cell carcinoma (2.5%), breast cancer (2%), thyroid cancer (1.8%) and glioma (1.8%). Taking into consideration previous studies that have investigated the landscape of germline LGRs in breast and ovarian cancer, we conclude that our cohort contains a similar proportion of *BRCA1/2* LGRs as was previously reported [8, 23, 26, 27].

Similar to other pathogenic germline SNVs/InDels, we observed the largest proportion of germline LGRs to occur on the DDR and HR pathways. Another pathway with high germline LGR prevalence is the FA pathway, which is known for causing a genetic disorder of the same name. Prior review has associated FA pathway functions with genomic stability, in addition to important tumor suppressing functions [28]. This may explain why samples in the LGR group have both a significantly higher chromosomal instability and a higher MSI‐H ratio than the SNV/InDel group.

Similar to most of the germline SNVs/InDels which have co‐occurrence with somatic mutations are MMR genes, germline LGRs in *MSH2* gene also has co‐occurrence with somatic mutations, which indicates that germline MMR‐deficiency (either germline SNV/InDel or LGR in MMR genes) might lead to elevated somatic mutation hits (Fig. 3A,B). *MLH1* and *MSH6* with LGRs might also have significantly co‐occurrent ratio with somatic mutations in a range of genes. However, due to the lack of enough patients with LGRs in *MLH1* and *MSH6* in the cohort, we cannot find an obvious correlation with MMR‐deficiency. Besides, germline LGRs in *TSC2* and somatic SNVs/InDels in *CDKN2* display significant trend of co‐occurrences, which is not shown in germline SNVs/InDels.

Our study has some limitations. Primarily, our automated approach to annotate pathogenic SNVs/InDels lack high reliability. However, this was remedied by including a step of manual review, which helped identify false positive variant calls.

## 5 Conclusions

In this study, we demonstrated the prevalence of germline LGRs in a pan‐cancer scale, beyond breast and ovarian cancer. We surveyed alterations in 15 659 patients from across 22 cancer types to identify and profile novel disease relevant germline LGRs. Patients with germline LGRs have significantly higher CIN and MSI‐H than both patients with germline SNVs/InDels and patients without germline mutations. Germline LGRs are more dispersive in multiple pathways (DDR, HR, cell cycle, MMR and PI3K) comparing to germline SNVs/InDels, which are mostly gathered in DDR and HR pathways. In the top 30 genes with germline LGRs, 4 genes (*RB1*, *MSH2*, *FANCA* and *PMS2*) have significantly higher ratio of germline LGRs rather than germline SNVs/InDels. Germline LGRs in *MSH2* gene has co‐occurrence with many somatic mutations. Also, germline LGRs in *TSC2* has co‐occurrence with somatic SNVs/InDels in *CDKN2*. While germline SNVs/InDels of *BRCA1* and *ATM* aggregate on hotspots of certain exons, their germline LGRs do not develop on certain exons, neither do LGRs of any other genes with high LGR prevalence. From this study, the profiles of the pathogenic/likely pathogenic germline alterations will fuel further investigations and highlight new understanding of LGRs across multiple cancer types.

## Author contributions

ZS, CB, MS, HT, Xiaoying Wu, and YW: visualization, formal analysis, and writing—Original Draft; XL: data analysis; BX, HB, Xue Wu, and YS: investigation, supervision, validation, project administration, and writing—review & editing. All authors read and approved the final manuscript.

## Conflict of interest

Authors Haimeng Tang, Xiaoying Wu, Yue Wang, Hua Bao, Xunbiao Liu, Xue Wu, Yang Shao are employees of Nanjing Geneseeq Technology Inc. All remaining authors declare no conflicts of interest.

### Peer review

The peer review history for this article is available at https://www.webofscience.com/api/gateway/wos/peer‐review/10.1002/1878‐0261.13430.

## Supporting information


**Fig. S1.** Validation of LGR detection method using ddPCR. A) Seven samples with *BRCA1/2* LGR mutations and eight samples with *RB1* LGR mutations were used to compare between NGS and copy number sensitive detection methods. B) Calculation for accuracy of NGS detection compared to ddPCR for each gene. Column and row names in the initial 2 x 2 contingency table represent all combinations of method and outcome (i.e., “NGS+” indicates that LGR was detected using targeted NGS. “ddPCR‐” means number of exons without LGRs as detected by ddPCR). Unit of counting is in exons. IGV window of rearranged regions in C) *BRCA1*, D) *BRCA2*, E) *RB1* (Chr 13: 49,053,847 – 49,053,886), and F) *RB1* (Chr 13: 48,954,220 – 48,954,259).

**Fig. S2.** Mutation count per exon or intron on genes with increased LGR proportion. Distribution of LGR and SNV/InDel counts on all exons and certain introns of A) *BRCA1*, B) *MSH2*, C) *FANCA*, and D) *ATM*.

**Fig. S3.** Comparisons of TMB, CIN, WGD, MSI and mutational signatures between patients with germline mutations in MRR genes. Same as A‐D in Figure 4, except only patients with germline mutations on MRR genes were included in each group of patients.

**Fig. S4.** Comparisons of TMB, CIN, WGD, MSI and mutational signatures between patients without germline mutations in MMR genes. Same as A‐D in Figure 4, except patient with germline mutations in MMR genes were excluded in each group of patients.

**Fig. S5.** Comparison of sample level metrics for patients with genes experiencing double‐hit events. Examined metrics include A) total mutation count, B) TMB, C) CIN, and D) WGD. TMB is defined as the number of non‐synonymous mutations per million bases of sequence. E) Proportion of samples in each group with each mutational signature. Comparisons between groups were made with the Wilcoxon ranked‐sum test and significance was determined at *P* < 0.05.

**Table S1.** Comparisons of clinical characteristics between patients grouped by pathogenic germline mutations.

## Data Availability

The dataset analyzed during the current study are available from the corresponding author on reasonable request.
