# Supplementary figures and images for: Comprehensive profiling of pathogenic germline large genomic rearrangements in a pan‐cancer analysis
Source: Mol Oncol. 2023 Apr 12;17(9):1917–29. doi: 10.1002/1878-0261.13430 (PMC10483597; doi:10.1002/1878-0261.13430)

A

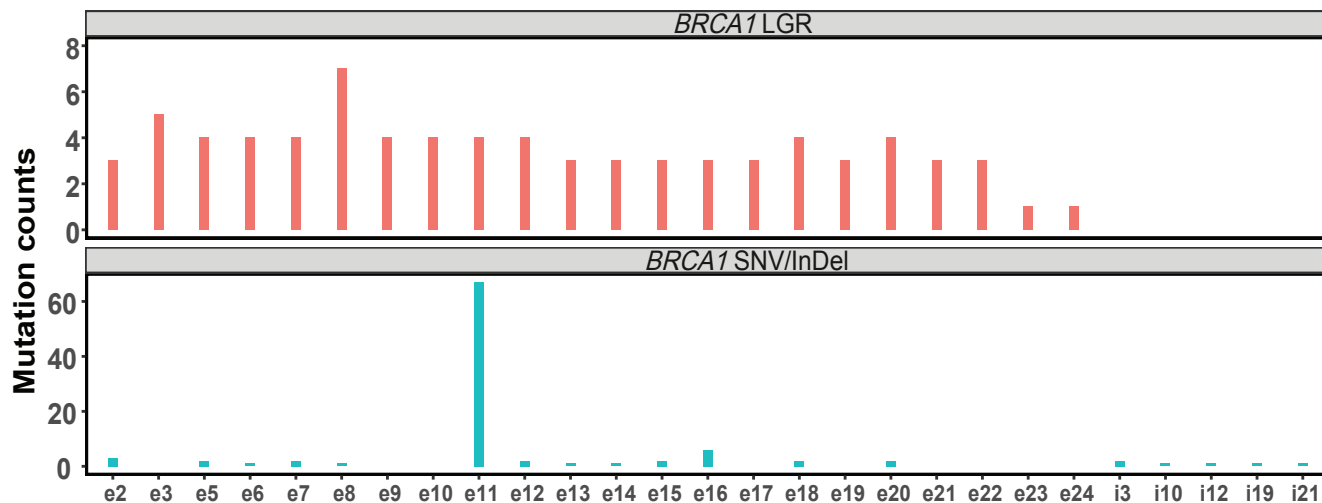

B

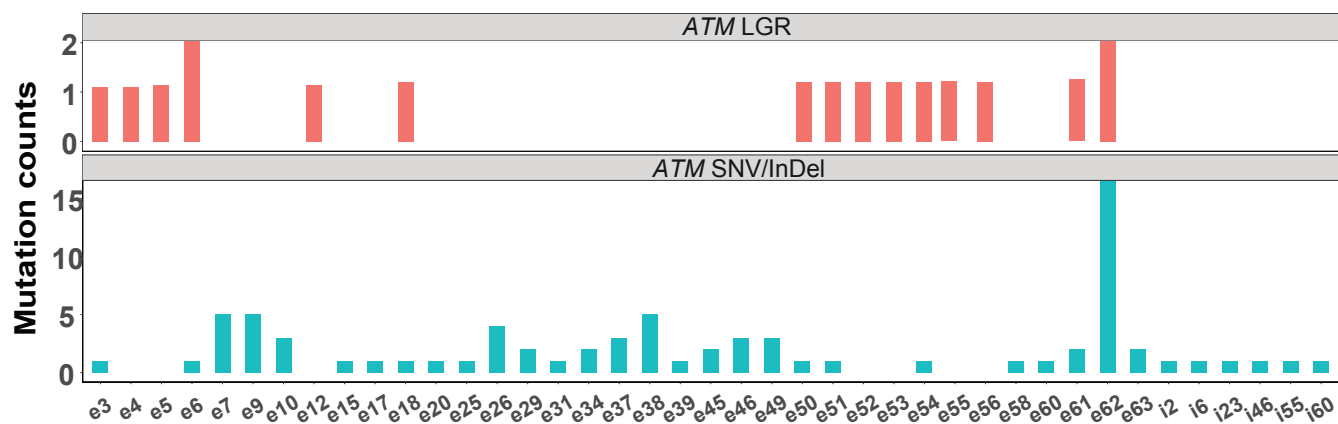

C

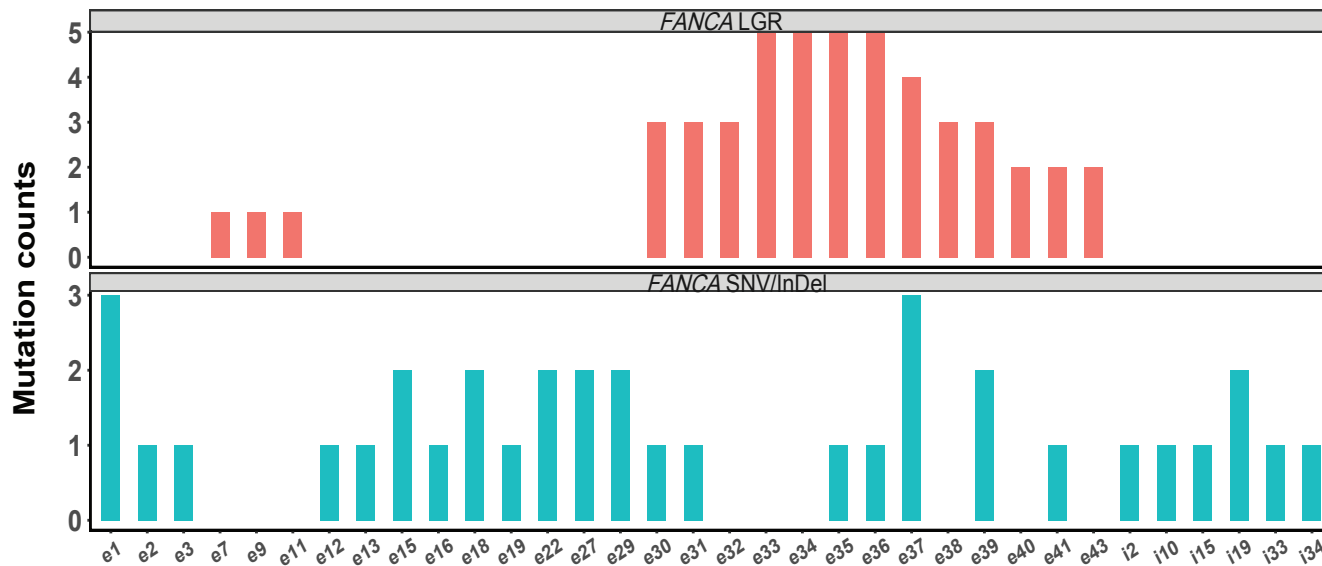

D

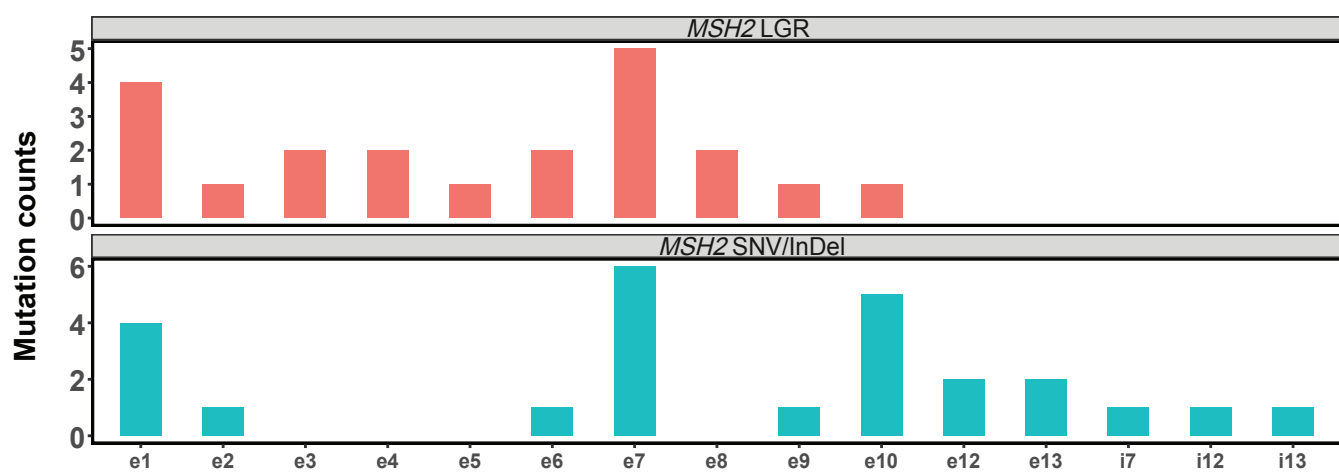

Supplement: Supplementary file 2 — Fig. S2. Mutation count per exon or intron on genes with increased LGR proportion. Distribution of LGR and SNV/InDel counts on all exons and certain introns of A) BRCA1, B) MSH2, C) FANCA, and D) ATM. [file MOL2-17-1917-s002.pdf]

A

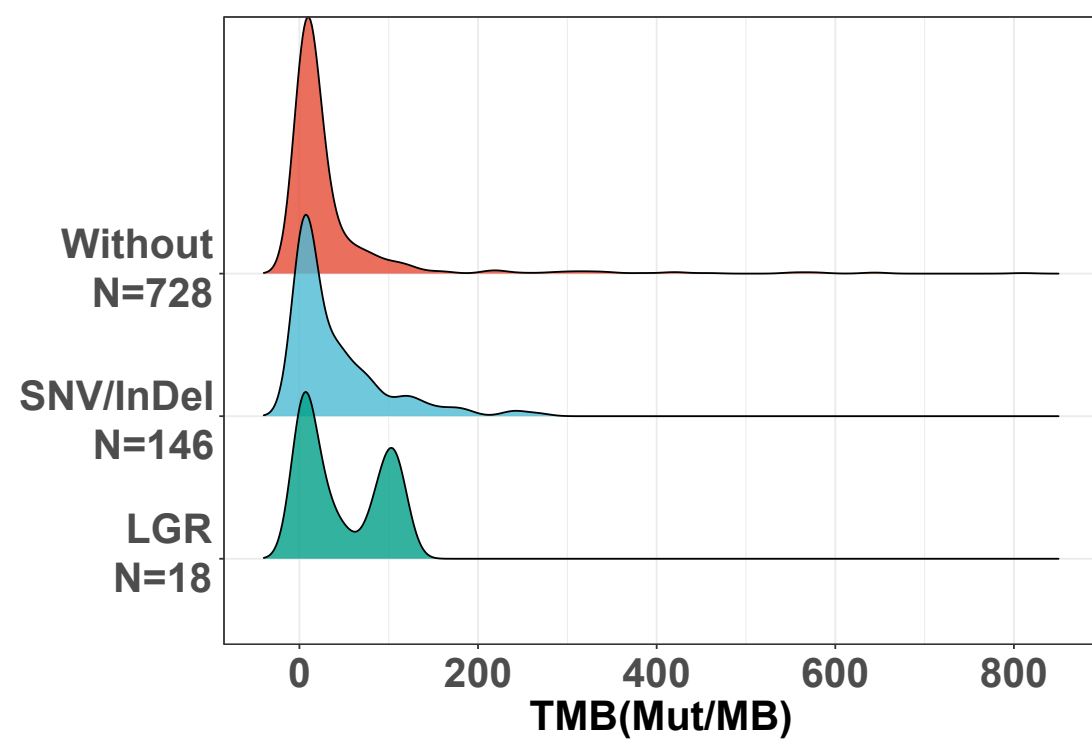

B

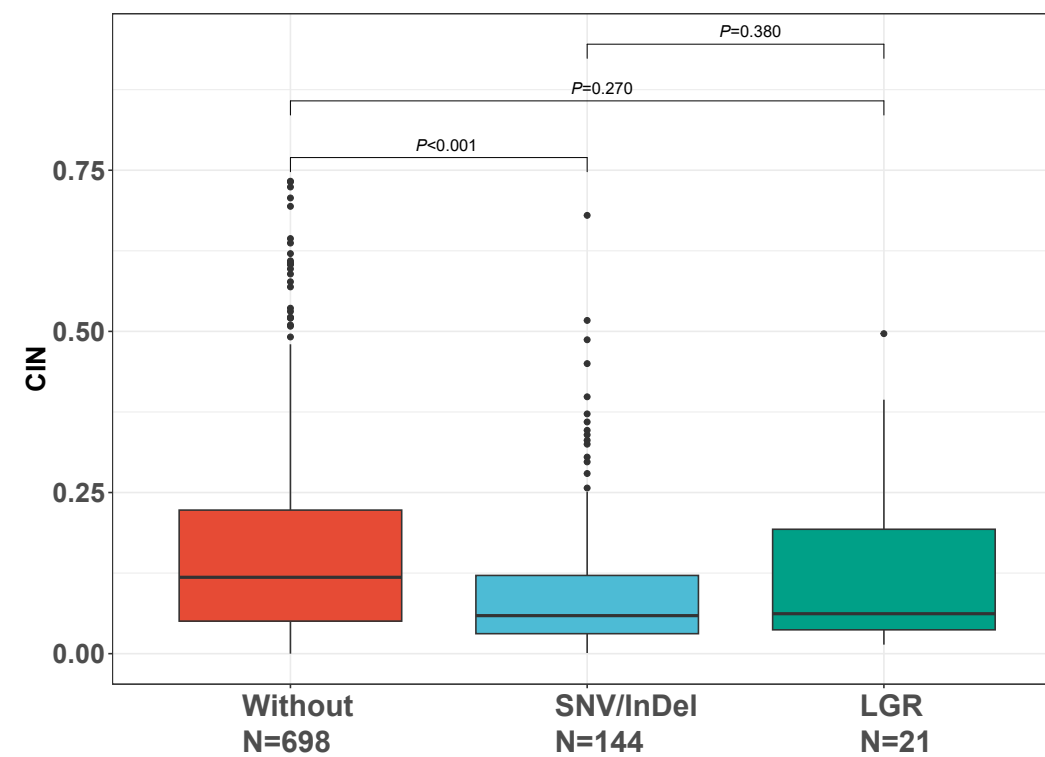

C

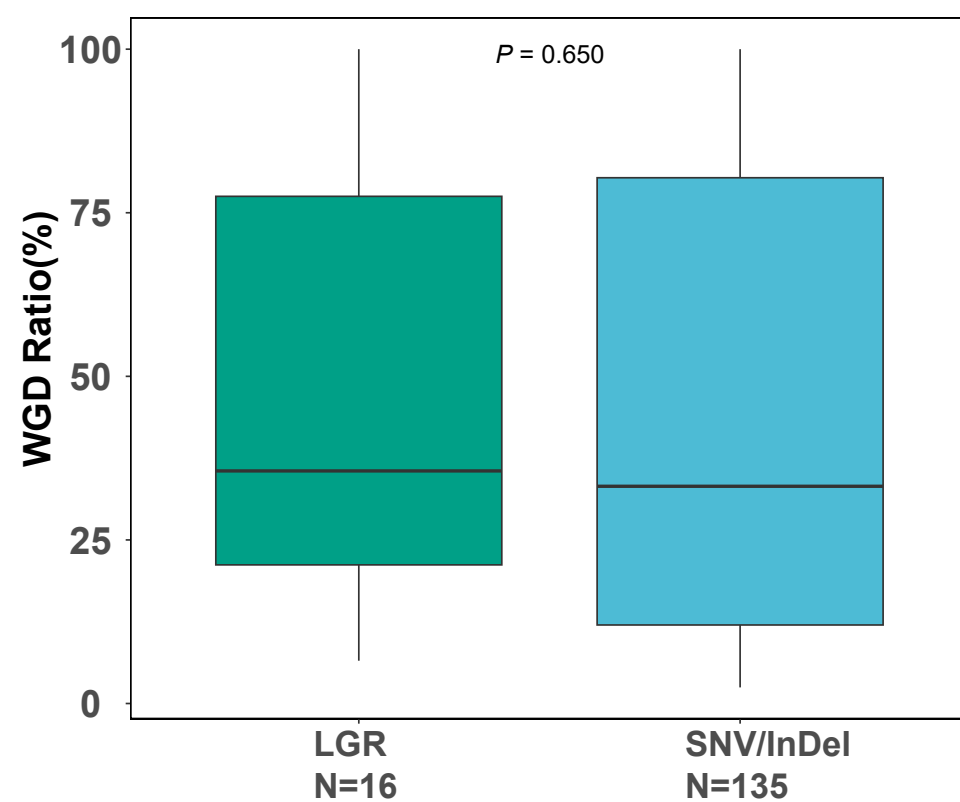

D

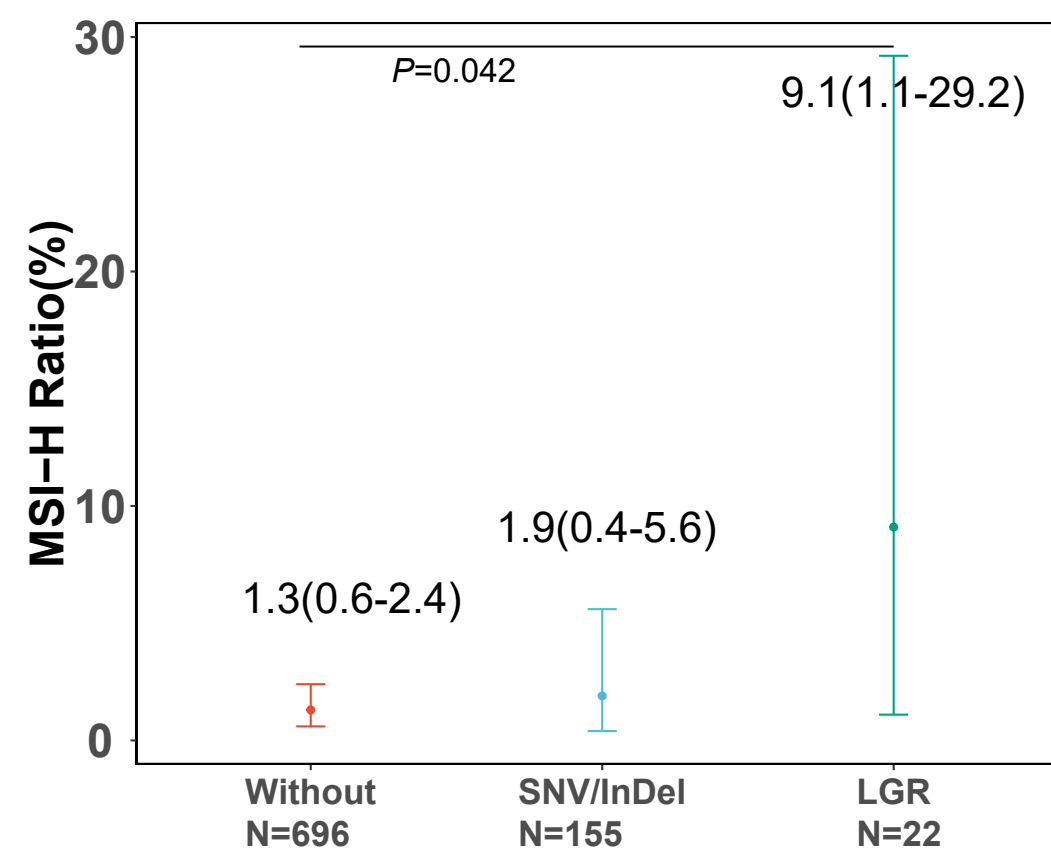

E

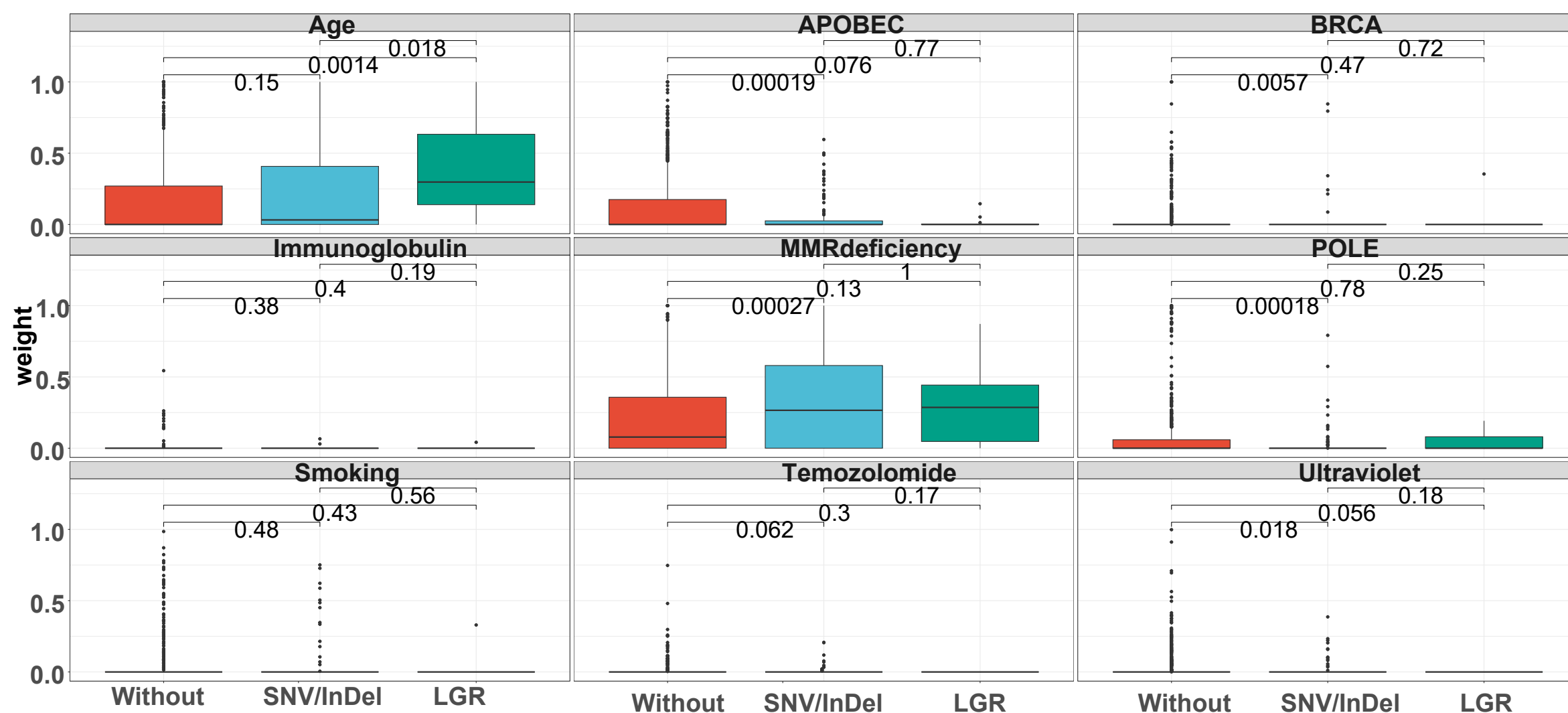

Supplement: Supplementary file 3 — Fig. S3. Comparisons of TMB, CIN, WGD, MSI and mutational signatures between patients with germline mutations in MRR genes. Same as A‐D in Figure 4, except only patients with germline mutations on MRR genes were included in each group of patients. [file MOL2-17-1917-s003.pdf]

A

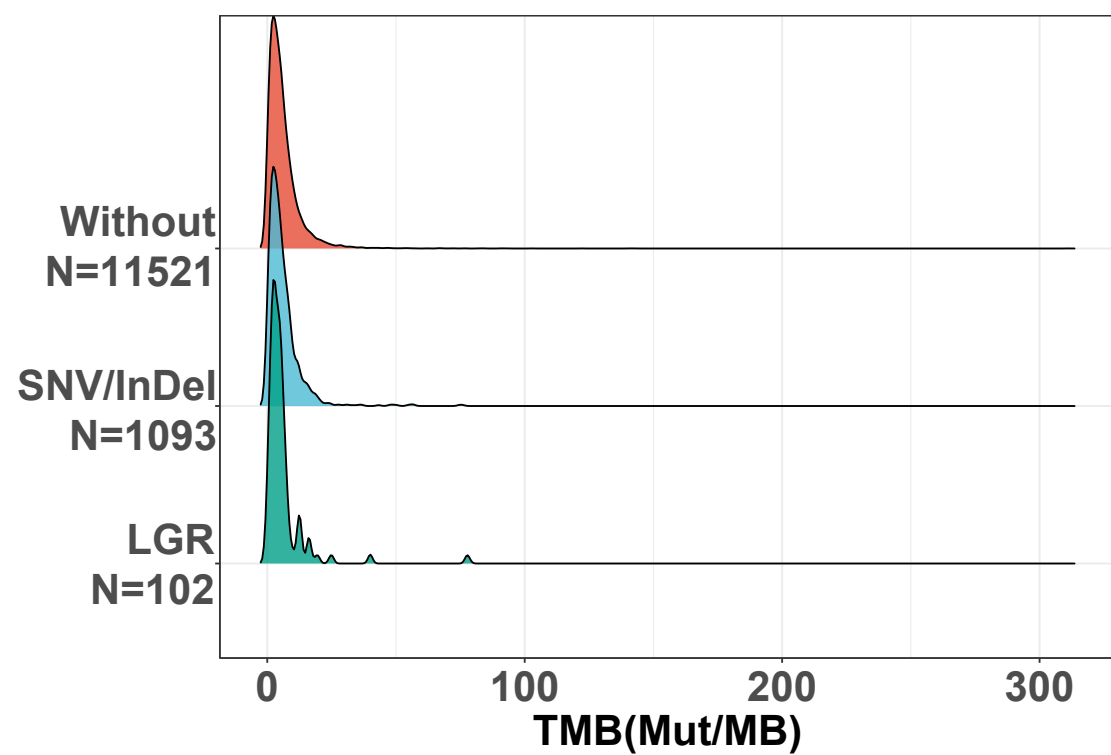

B

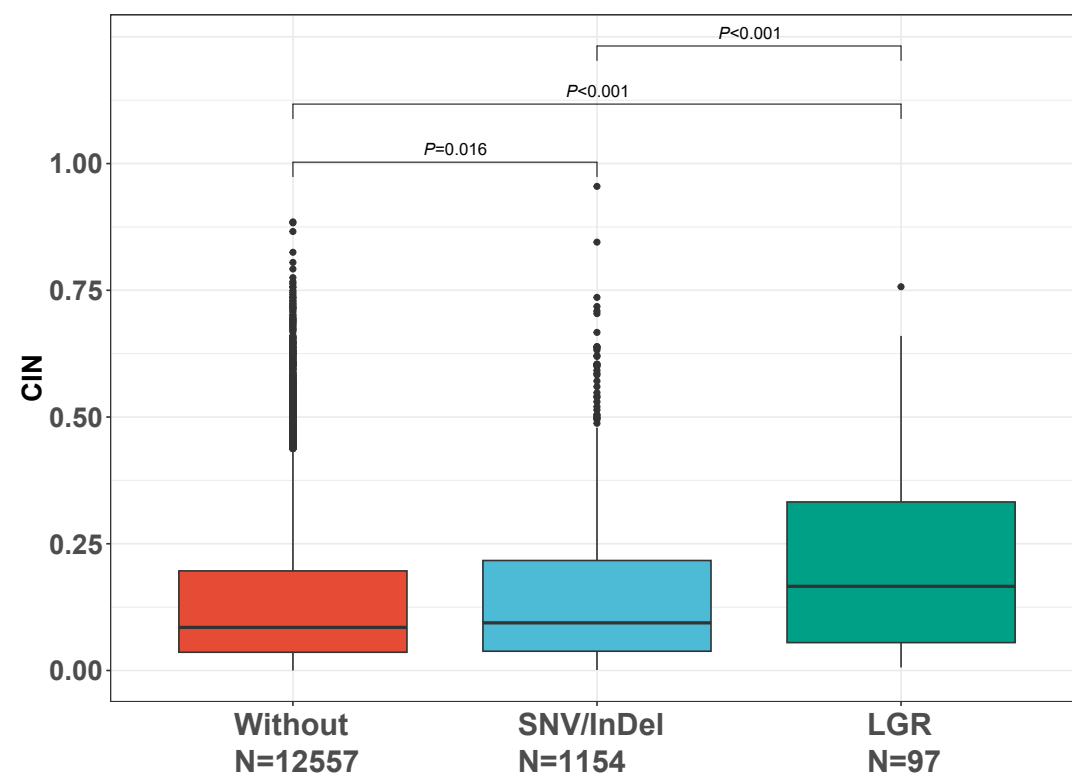

C

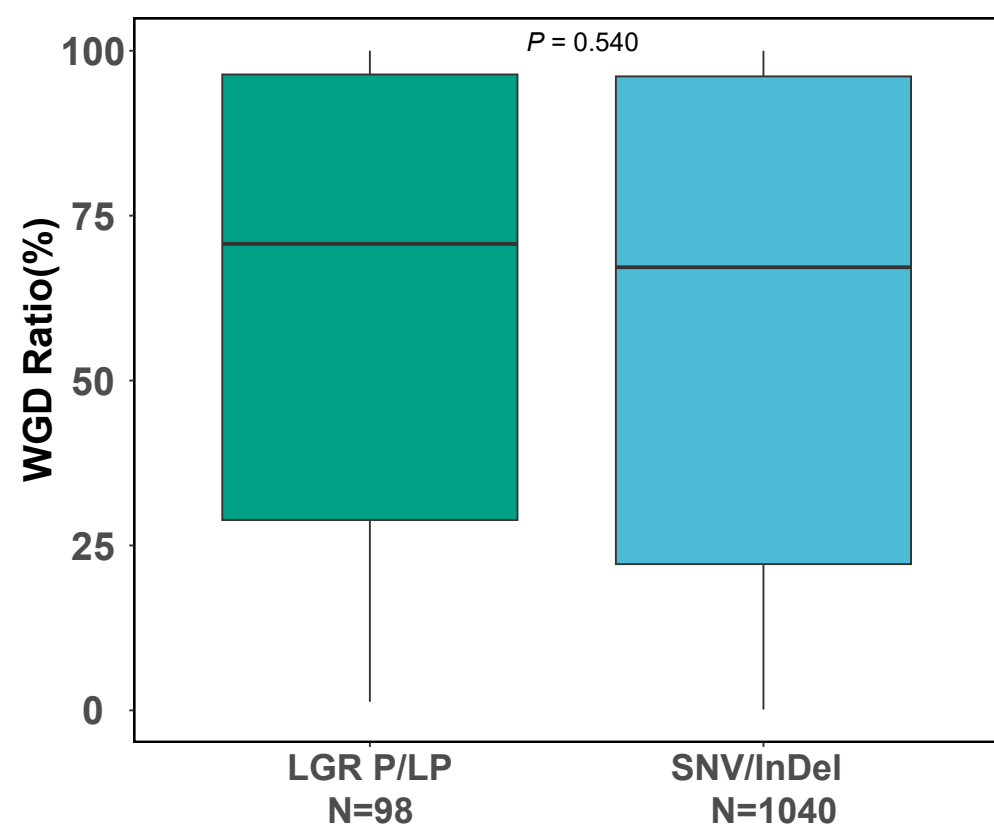

D

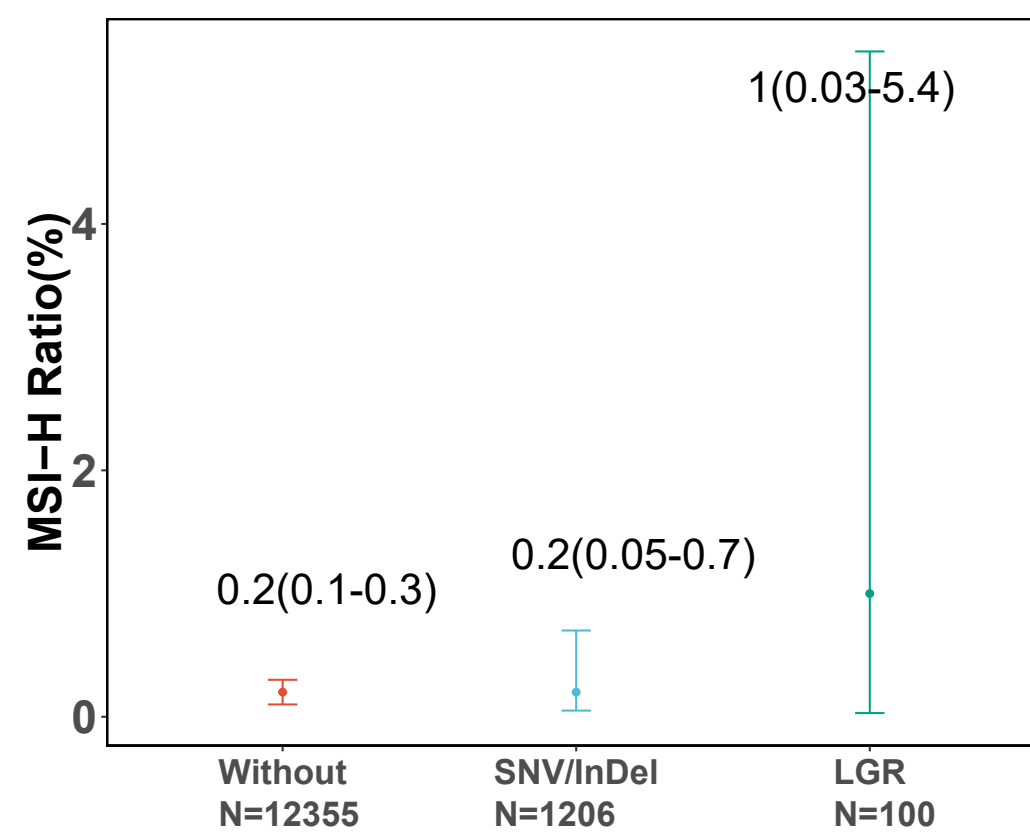

E

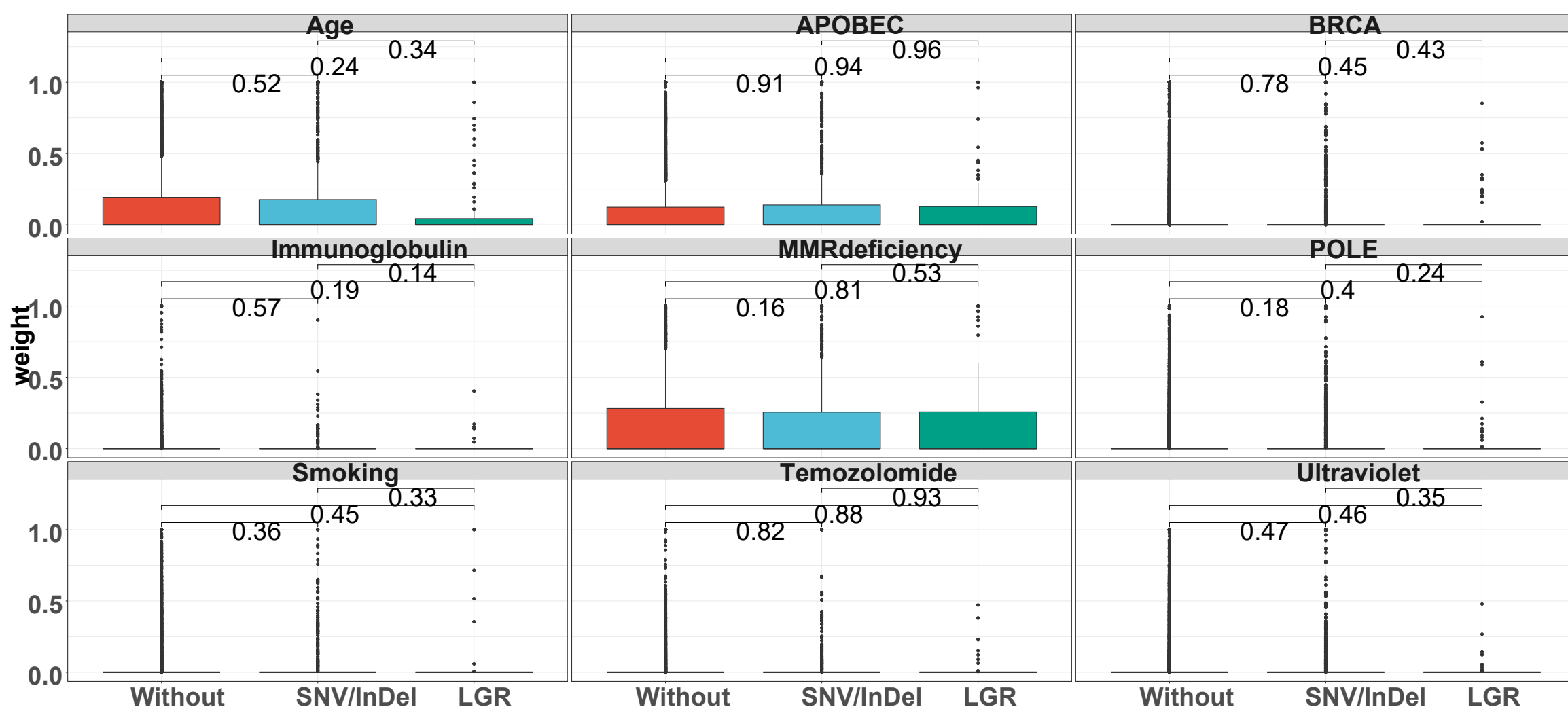

Supplement: Supplementary file 4 — Fig. S4. Comparisons of TMB, CIN, WGD, MSI and mutational signatures between patients without germline mutations in MMR genes. Same as A‐D in Figure 4, except patient with germline mutations in MMR genes were excluded in each group of patients. [file MOL2-17-1917-s001.pdf]

A

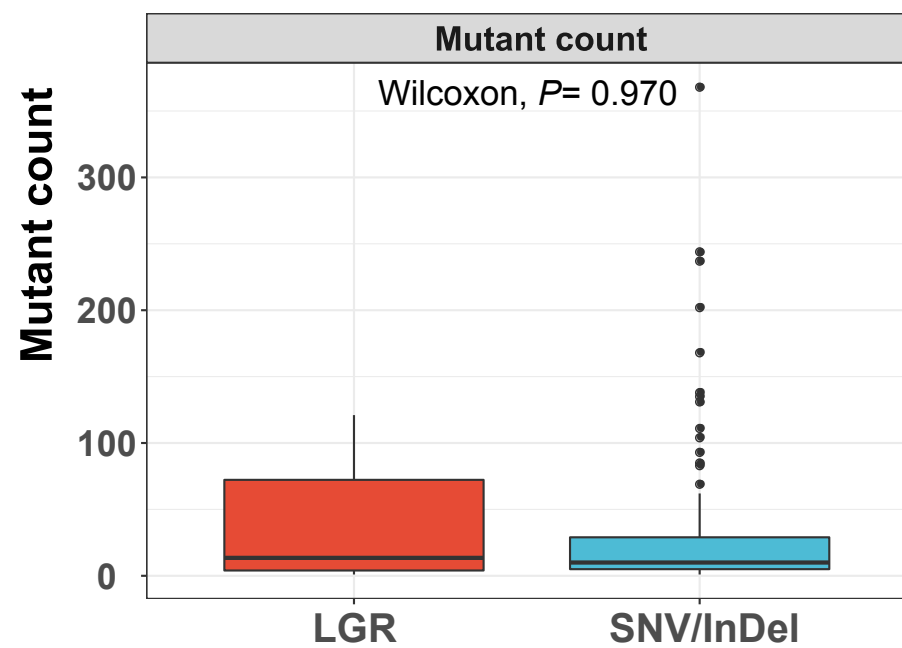

B

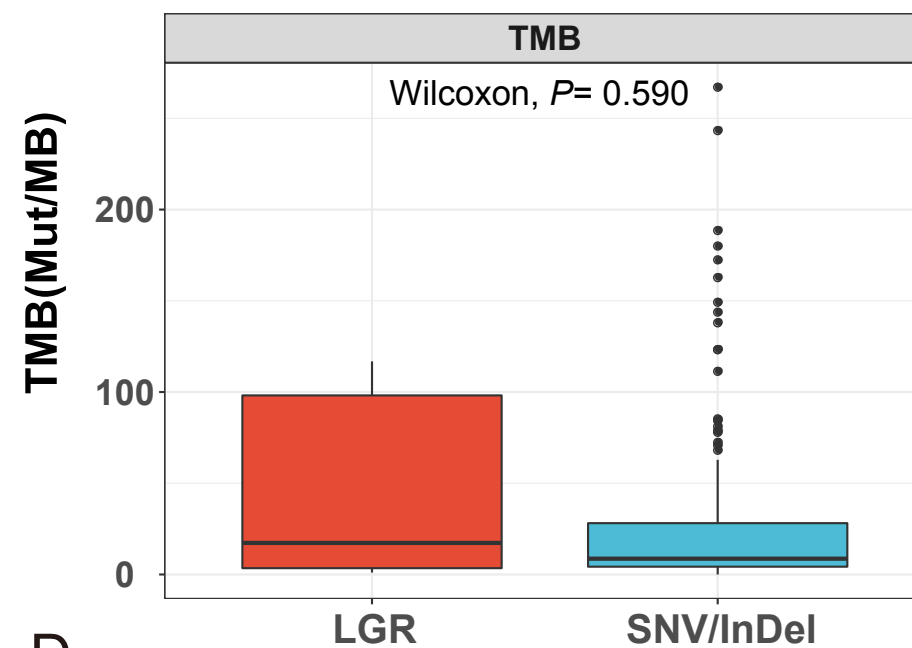

C

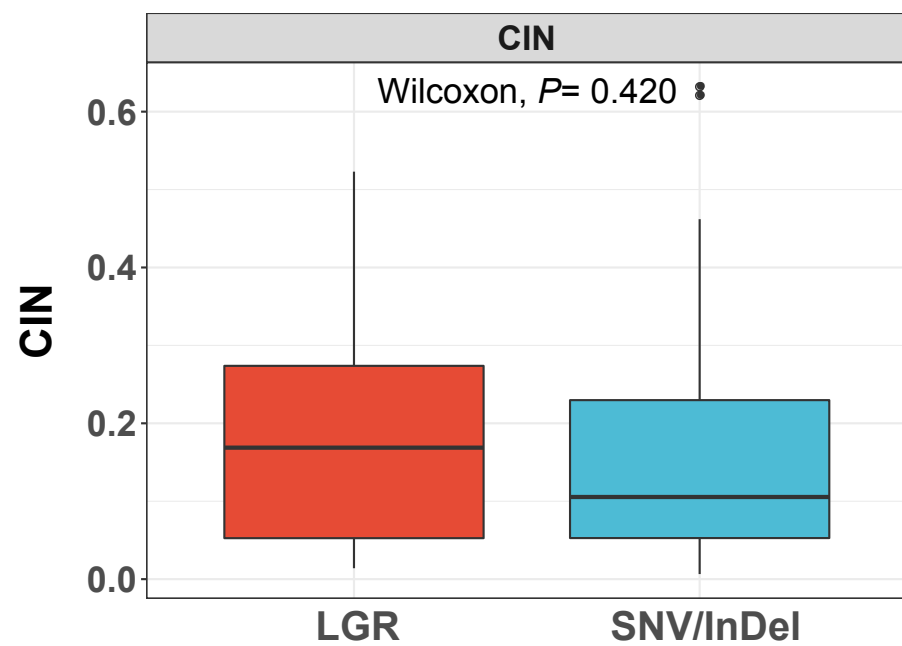

D

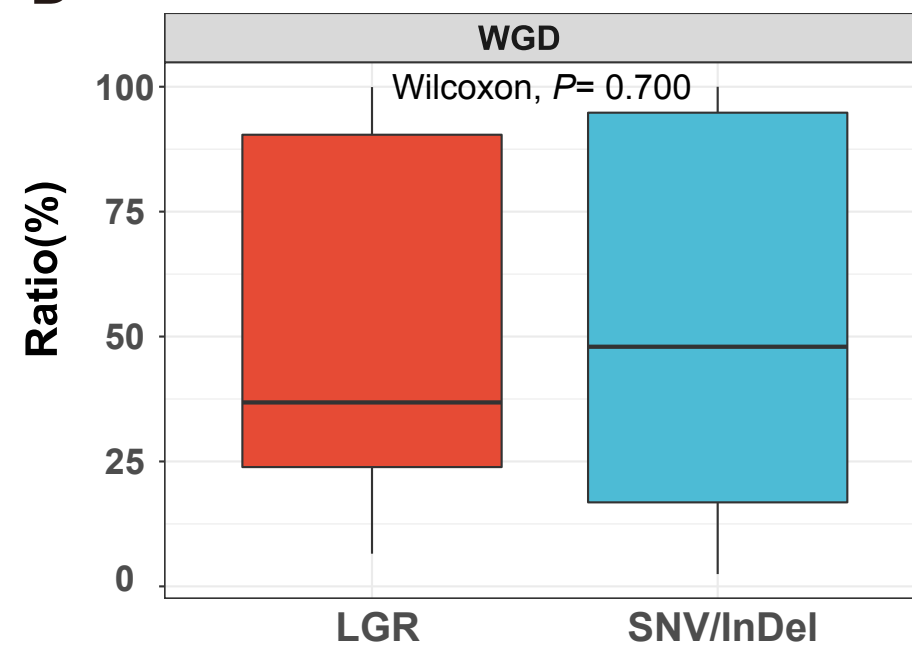

E

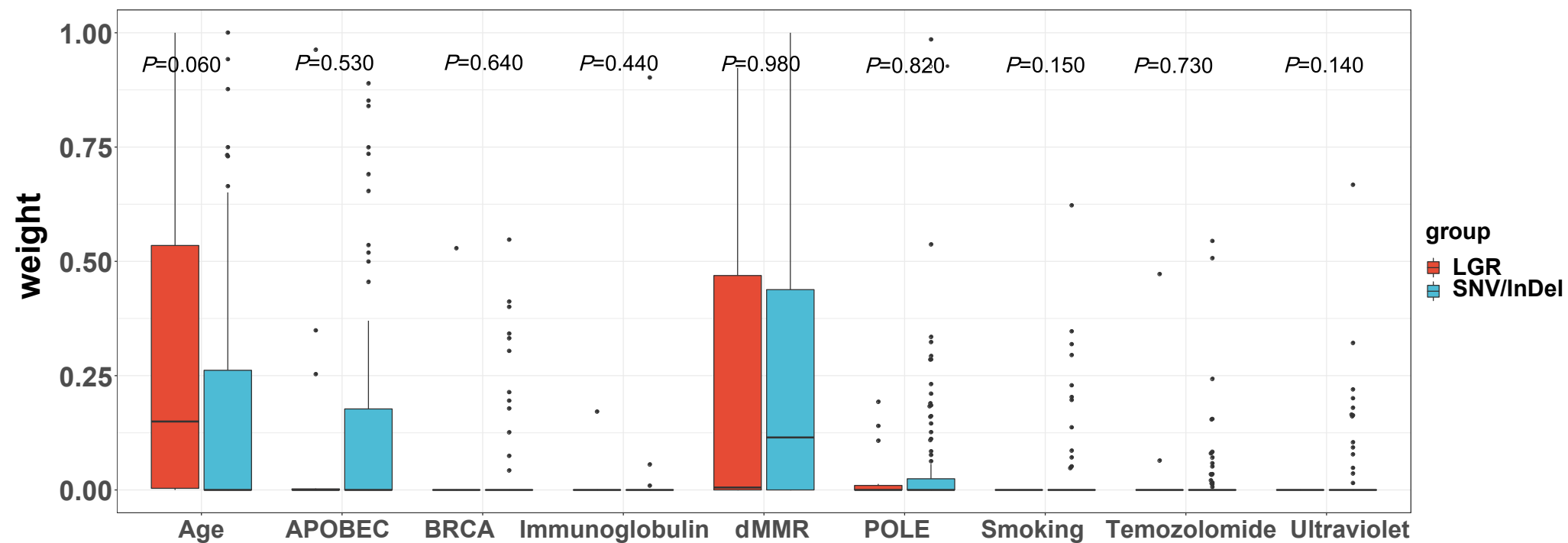

Supplement: Supplementary file 5 — Fig. S5. Comparison of sample level metrics for patients with genes experiencing double‐hit events. Examined metrics include A) total mutation count, B) TMB, C) CIN, and D) WGD. TMB is defined as the number of non‐synonymous mutations per million bases of sequence. E) Proportion of samples in each group with each mutational signature. Comparisons between groups were made with the Wilcoxon ranked‐sum test and significance was determined at P < 0.05. [file MOL2-17-1917-s005.pdf]
