# Supplementary material for: Comprehensive profiling of pathogenic germline large genomic rearrangements in a pan‐cancer analysis
Source: Mol Oncol. 2023 Apr 12;17(9):1917–29. doi: 10.1002/1878-0261.13430 (PMC10483597; doi:10.1002/1878-0261.13430)

A

ID	NGS-*BRCA1*	ddPCR-*BRCA1*	NGS-*BRCA2*	ddPCR-*BRCA2*
B1	del e2-22	del e2-e22	del e8	wt
B2	del e8	del e8	wt	wt
B3	wt	wt	del e20-e21; e23-e24	del e23-e24
B4	del e2-12	del e2-e12	wt	wt
B5	gain:e2-12	gain:e2-e12	wt	wt
B6	del e8	del e8	wt	wt
B7	del e8-e9; e13-e14	del e8-e9	del e2-8; e12-27	wt

ID	NGS-*RB1*	ddPCR-*RB1*
R1	del e21-26	del e21-26
R2	negative	del e20
R3	del e1-2; e7-27	del e1-27
R4	del e1-27	del e1-27
R5	del e13-16	del e13-16
R6	del e18	del e18
R7	del e1-27	del e1-27
R8	del e2-27	del e1-27

B

BRCA1-exons N=161 *BRCA1*:NM_007294

	NGS+	NGS-	Sensitivity	Specificity	Accuracy
ddPCR+	47	0	100.00%	98.25%	98.76%
ddPCR-	2	112			

BRCA2-exons N=196 *BRCA2*:NM_000059

	NGS+	NGS-	Sensitivity	Specificity	Accuracy
ddPCR+	2	0	100.00%	86.60%	86.73%
ddPCR-	26	168			

RB1-exons N=216 *RB1*:NM_000321

	NGS+	NGS-	Sensitivity	Specificity	Accuracy
ddPCR+	114	6	95.00%	100.00%	97.22%
ddPCR-	0	96			

C

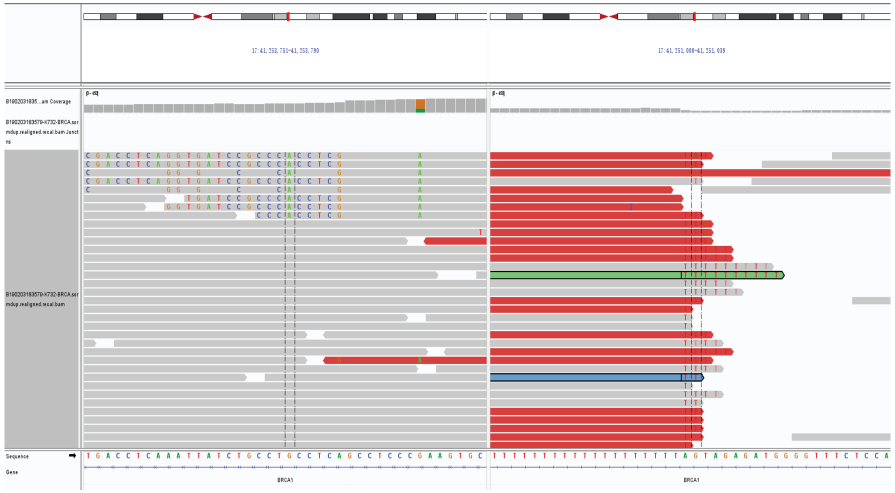

D

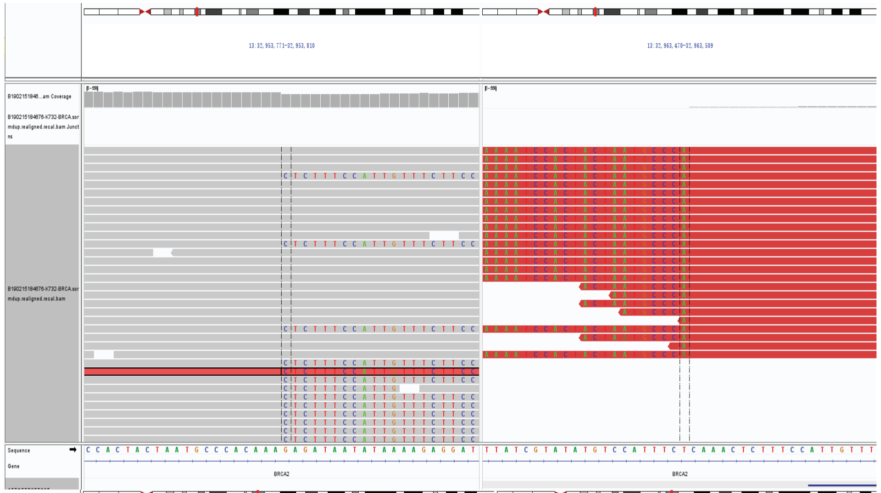

E

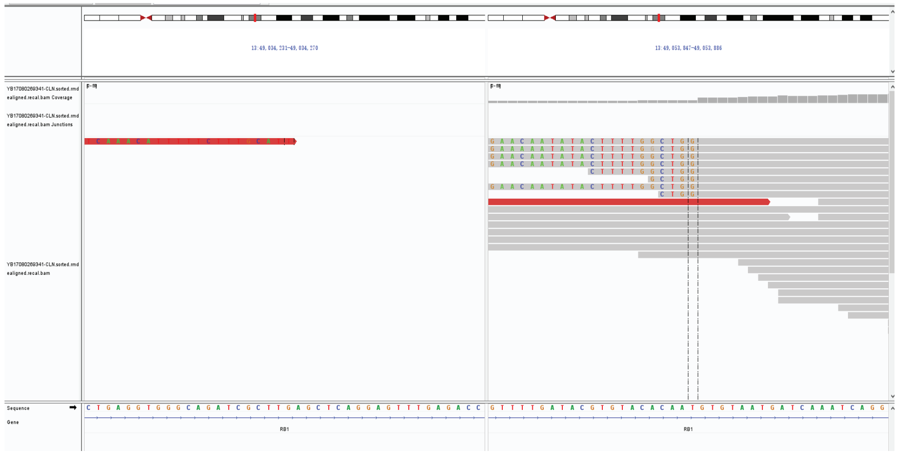

F

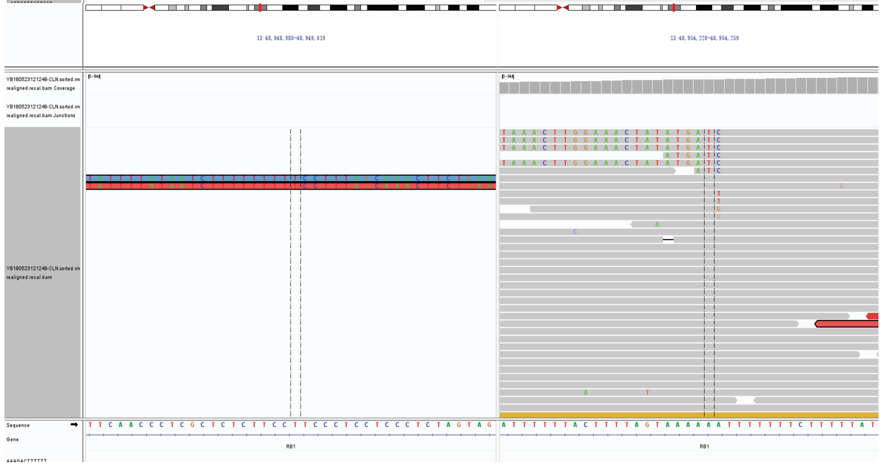

Supplement: Supplementary file 1 — Fig. S1. Validation of LGR detection method using ddPCR. A) Seven samples with BRCA1/2 LGR mutations and eight samples with RB1 LGR mutations were used to compare between NGS and copy number sensitive detection methods. B) Calculation for accuracy of NGS detection compared to ddPCR for each gene. Column and row names in the initial 2 x 2 contingency table represent all combinations of method and outcome (i.e., “NGS+” indicates that LGR was detected using targeted NGS. “ddPCR‐” means number of exons without LGRs as detected by ddPCR). Unit of counting is in exons. IGV window of rearranged regions in C) BRCA1, D) BRCA2, E) RB1 (Chr 13: 49,053,847 – 49,053,886), and F) RB1 (Chr 13: 48,954,220 – 48,954,259). [file MOL2-17-1917-s004.pdf]
